# Inhibition of Pyk2 blocks lung inflammation and injury in a mouse model of acute lung injury

**DOI:** 10.1186/1465-9921-13-4

**Published:** 2012-01-18

**Authors:** Yingli Duan, Jonathan Learoyd, Angelo Y Meliton, Alan R Leff, Xiangdong Zhu

**Affiliations:** 1 Section of Pulmonary and Critical Care Medicine, Department of Medicine, The University of Chicago, Chicago, IL 60637; 2 Departments of Anesthesia and Critical Care, Neurobiology and Pediatrics, and Committees on Clinical Pharmacology, Cell Physiology and Molecular Medicine, Division of the Biological Sciences, The University of Chicago, Chicago, IL 60637

**Keywords:** inflammation, lipopolysaccharide, lung, neutrophils, Pyk2

## Abstract

**Background:**

Proline-rich tyrosine kinase 2 (Pyk2) is essential in neutrophil degranulation and chemotaxis in vitro. However, its effect on the process of lung inflammation and edema formation during LPS induced acute lung injury (ALI) remains unknown. The goal of the present study was to determine the effect of inhibiting Pyk2 on LPS-induced acute lung inflammation and injury in vivo.

**Methods:**

C57BL6 mice were given either 10 mg/kg LPS or saline intratracheally. Inhibition of Pyk2 was effected by intraperitoneal administration TAT-Pyk2-CT 1 h before challenge. Bronchoalveolar lavage analysis of cell counts, lung histology and protein concentration in BAL were analyzed at 18 h after LPS treatment. KC and MIP-2 concentrations in BAL were measured by a mouse cytokine multiplex kit. The static lung compliance was determined by pressure-volume curve using a computer-controlled small animal ventilator. The extravasated Evans blue concentration in lung homogenate was determined spectrophotometrically.

**Results:**

Intratracheal instillation of LPS induced significant neutrophil infiltration into the lung interstitium and alveolar space, which was attenuated by pre-treatment with TAT-Pyk2-CT. TAT-Pyk2-CT pretreatment also attenuated 1) myeloperoxidase content in lung tissues, 2) vascular leakage as measured by Evans blue dye extravasation in the lungs and the increase in protein concentration in bronchoalveolar lavage, and 3) the decrease in lung compliance. In each paradigm, treatment with control protein TAT-GFP had no blocking effect. By contrast, production of neutrophil chemokines MIP-2 and keratinocyte-derived chemokine in the bronchoalveolar lavage was not reduced by TAT-Pyk2-CT. Western blot analysis confirmed that tyrosine phosphorylation of Pyk2 in LPS-challenged lungs was reduced to control levels by TAT-Pyk2-CT pretreatment.

**Conclusions:**

These results suggest that Pyk2 plays an important role in the development of acute lung injury in mice and that pharmacological inhibition of Pyk2 might provide a potential therapeutic strategy in the pretreatment for patients at imminent risk of developing acute lung injury.

## Background

Acute lung injury (ALI), which may progress to Acute Respiratory Distress Syndrome (ARDS), is associated with high morbidity and mortality in critically ill patients [1,2]. Despite intense research and multiple diverse therapeutic trials, there still are few effective measures for prevention or treatment of ARDS. ARDS is a frequent complication that emerges in patients having sepsis. Lipopolysaccharides (LPS) components of endotoxin are responsible for the enhanced inflammatory response of ALI and ARDS [3]. The LPS- induced mouse model of ALI is associated with increased neutrophilic lung inflammation and endothelial barrier dysfunction [4-6]. Intranasal instillation of LPS stimulates airway epithelial cells to release proinflammatory cytokines and chemotactic factors, which causes subsequent neutrophilic infiltration and ultimately results in lung tissue injury [7]. This study was designed to determine whether inhibition of the protein tyrosine kinase Pyk2, which mediates a wide variety of cellular activities including cell migration [8], blocks neutrophil infiltration and lung injury induced by LPS in mice.

Protein tyrosine kinase Pyk2, a non-receptor tyrosine kinase structurally related to focal adhesion kinase (FAK) [8,9], is a common mediator of signaling by growth factors, integrins, and G-protein-coupled receptors. Pyk2 inhibition has been shown to decrease neutrophil chemotaxis, degranulation, and superoxide release in vitro [10-12]. Overexpression of dominant negative Pyk2 [11] or silencing Pyk2 expression [13] reduces chemotaxis of HL-60-derived neutrophils-like cells. A recent study demonstrated that Pyk2 is activated by non-muscle myosin light-chain kinase and mediates neutrophil transendothelial migration [14]. Previous in vivo studies have shown that recruitment of macrophages is attenuated in Pyk2-deficient mice after stimulation with chemokine and in response to carageenan [15]. Pyk2-deficient mice lack marginal zone B cells in the spleen. This has been associated with a decreased motility of B lymphocytes in response to a variety of chemokines [16]. Our laboratory recently has reported that TAT-Pyk2-CT, a fusion protein in which Pyk2 C-terminal domain (amino acid 680-1009) is fused to a cell-permeable TAT peptide, blocks eosinophilic airway inflammation and airway hyperresponsiveness in an ovalbumin- induced mouse model of asthma [17]. From these observations we have hypothesized that the Pyk2 signaling pathway also may play an important role in LPS-mediated lung inflammation and that inhibition of Pyk2 may reduce neutrophil infiltration in the lung and reduce lung injury in vivo.

The objective of this study was to define the anti-inflammatory effects of Pyk2 inhibition in a LPS-induced mouse lung injury model. Intranasal instillation of LPS into mice can produce a controlled ALI response without causing systemic inflammation and multi-organ failure and was therefore chosen for these studies [18]. We intratracheally administered LPS because this delivery avoids deposition in the nasal passages [19]. We found that TAT-Pyk2-CT blocked LPS-induced neutrophilic lung inflammation and vascular leakage without blocking MIP-2 and keratinocyte- derived chemokine (KC) production in LPS challenged lungs.

## Methods

### Murine model of ALI

Female C57BL/6 mice, aged 10-12 wk old, were maintained on standard laboratory chow ad libitum. Experimental protocols were approved by the University of Chicago IACUC Review Board. Anesthetized mice were instilled through a catheter inserted into the trachea with either saline solution or 10 mg/kg LPS [5] (Strain 055:B5, Sigma-Aldrich, St. Louis, MO). Animals were studied 18 h after administration of LPS. TAT-Pyk2-CT is a 50 kDa fusion protein in which the TAT peptide is fused to the N terminus of the proline-rich C-terminal domain of Pyk2. TAT-Pyk2-CT acted as a cell membrane permeable inhibitor of Pyk2 that blocked both Pyk2 binding to its C-terminal associated proteins (p130^cas^, paxillin), as well as tyrosine phosphorylation of endogenous Pyk2 [12,20]. TAT-GFP is a ~35 kDa fusion protein, which was used as a control for TAT-Pyk2-CT. The purification of these TAT fusion proteins has been described previously in detail [17,20]. TAT-Pyk2-CT at 10 mg/kg was given by intraperitoneal injection 1 h prior to LPS administration, and control mice received an equal amount of TAT-GFP before LPS challenge.

### Lung morphology and histology

Mouse lungs were inflated to 25 cm H_2_O with 1% paraformadehyde, embedded in paraffin, stained with hematoxylin-eosin (H&E) and observed by light microscopy. All lung fields at ×20 magnification were examined for each sample. Lungs were photographed prior to histological analysis.

### Analysis of BAL fluid

Bronchoalveolar lavage (BAL) was performed by injecting 3× with 0.8 ml of PBS into the lung and gently aspirating the fluid. The BAL fluid samples were centrifuged to obtain the cell pellet. Cytoslides were prepared and stained with Diff-Quick (Dade Diagnostics, Deerfield, IL), and cell counts were determined using a hemocytometer. Supernatants were stored for subsequent analysis of protein and cytokine contents.

### Vascular leakage

To assess vascular leakage, Evans blue dye (30 mg/kg) was injected into the external jugular vein 2 h before the termination of the experiment as described [21]. Evans blue dye has a very high binding affinity for serum albumin. When the vascular barrier in the lung is compromised, albumin-bound Evans blue moves into the lung parenchyma. The lungs were perfused free of blood with phosphate-buffered saline containing 5-mM ethylenediaminetetraacetic acid via thoracotomy, excised *en bloc*, blotted dry, weighed, and snap frozen in liquid nitrogen. The right lung was homogenized in PBS (1 ml/100 μg tissue), incubated with 2 volumes of formamide to extract the dye (18 h, 60°C), and centrifuged at 5,000 × *g *for 30 minutes, and the optical density of the supernatant was determined spectrophotometrically at 620 nm. The extravasated EB concentration in lung homogenate was calculated against a standard curve and was expressed as micrograms of Evans blue dye per gram of lung. The vascular leakage was also determined by measurement of total protein concentration in supernatants of BAL using Bradford assay (Bio-Rad, Hercules, CA).

### Determination of total lung myeloperoxidase (MPO) content

Neutrophil parenchymal infiltration, as reflected by MPO activity, was measured as described [5]. The mouse lungs were homogenized in 0.8 ml PBS containing 1% Triton X-100 and centrifuged for 10 min at 10,000 *g *at 4°C. The supernatants were collected and MPO activity was measured. Briefly, 50 μl supernatants from lung homogenates were added to 100 μl of substrate solution (0.01% H_2_O_2_, 0.167 mg/mL *O*-dianizidine dihydrochloride, and 0.5% hexadecyltrimethylammonium bromide in 50 mM potassium phosphate buffer, pH 5.5). The reaction mixture was terminated by addition of 50 μl of sulfuric acid; absorbance was measured at 405 nm in a microplate reader (Thermomax; Molecular Devices, Menlo Park, CA).

### Measurement of transthoracic static compliance

Transthoracic static compliance was measured in vivo 18 h after saline or LPS challenge. The intubated animal was connected to a computer-controlled small animal ventilator (Scireq, Vancouver, Canada) that delivered a tidal volume of 6 ml/kg at a frequency of 120 breaths/min [22]. Lungs were inflated with 30 cm H_2_O transthoracic pressure, and volume was gradually decreased in 0.1-ml increments. The static lung compliance was determined by recording the lung pressure change associated with each volume change in the system when the trachea was occluded to ambient air. Two full inflation-deflation cycles were determined for each mouse to achieve a volume history, and the pressure-volume characteristics of the lungs were generated.

### Measurement of BAL cytokines/chemokines

A mouse cytokine multiplex kit (Mouse Cytokine multiplex; Millipore) was used to assay BAL fluid for KC and MIP-2 according to the manufacturer's instructions.

### Western blot analysis of the tyrosine phosphorylation of Pyk2

To examine the efficacy of TAT-Pyk2-CT in inhibiting Pyk2 activation in the lungs, TAT-Pyk2-CT (10 mg/kg) or TAT-GFP control was administered i.p. 1 h before i.t administration of LPS. The lungs were excised at 18 h after LPS challenge, rinsed in PBS, blotted dry, snap frozen in liquid nitrogen, and stored at -80°C. For preparation of whole-lung cell extracts, 10 mg of ground lung tissue was resuspended in 500 μl of lysis buffer (10 mM Tris, pH 7.4, 5 mM MgCl_2_, 50 U/ml DNase and RNase, 10 μg/ml each of leupeptin, aprotinin and pepstatin, 1 mM PMSF, and then homogenized for 30 s at a speed of 15,000 rpm in an Ultra-Turrax homogenizer). After a 30-min incubation on ice, homogenates were centrifuged at 16,000 *g *for 10 min. Protein concentrations were determined using the Bradford assay, the supernatants were mixed with Laemmli sample buffer, and boiled for 5 min. The samples were then used for analyzing Pyk2 tyrosine phosphorylation.

Samples were subjected to SDS-PAGE, using 7.5% acrylamide gels under reducing condition (15 mA/gel). Electrotransfer of proteins from the gels to nitrocellulose membrane was achieved using a semi-dry system (400 mA, 60 min). The membrane was blocked with 1% BSA for 60 min, then incubated with 1:1,000 anti-Pyk2 or anti-Y^402 ^phospho-Pyk2, diluted in Tris-buffered saline plus 0.05% Tween 20 (TBS-T) overnight. The membranes then were washed three times for 20 min with TBS-T. Anti-rabbit IgG conjugated with horseradish peroxidase was diluted 1:3000 in TBS-T and incubated with nitrocellulose membrane for 60 min. The membrane was again washed three times with TBS-T and assayed by an enhanced chemiluminescence system (Amersham, Arlington Heights, IL). Western blots were analyzed by Bio-Rad GS-710 densitometer and optical density (OD) data were expressed as the ratio of phospho-Pyk2 to total Pyk2.

### Data analysis

All values were expressed as means ± SEM. When more than two groups were compared, differences among the groups were determined by one-way ANOVA followed by Fisher's least significant difference test.

## Results

### Effect of TAT-Pyk2-CT on Pyk2 activation in vivo

**Figure 1:**
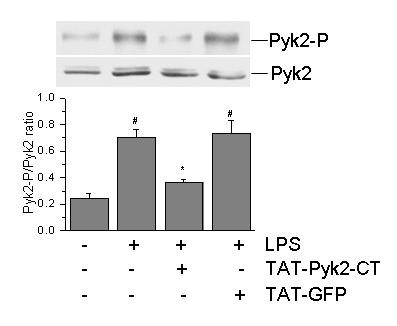
**Inhibition of tyrosine phosphorylation of Pyk2 by TAT-Pyk2-CT in lung tissue**. Mice were treated intraperitoneally with 10 mg/kg TAT-Pyk2-CT or TAT-GFP 1 h prior to LPS challenge. Mice were sacrificed 18 h after LPS challenge and the lungs were removed. Lung lysates were analyzed for phosphorylated Pyk2 at Y402 using Western blot analysis. Upper panel is the representative immunoblot from three experiments. The lower panel is the relative optical density of phosphorylated Pyk2 normalized against corresponding total Pyk2 from three blots. #*P *< 0.05 vs. saline control; *P < 0.05 *vs*. LPS alone group.

LPS challenge caused substantially increased tyrosine phosphorylation of Pyk2 compared to saline control (Figure 1). Lungs obtained from TAT-Pyk2-CT-treated and LPS challenged mice showed significantly reduced amounts of phosphorylated Pyk2, indicating the inhibition of Pyk2 activation by TAT-Pyk2-CT. By contrast, levels of phosphorylated Pyk2 in lungs obtained from LPS challenged mice pretreated with TAT-GFP control were comparable to mice receiving LPS treatment alone. The normalized optical density of phosphorylated Pyk2 immunostaining in saline controls was 0.25 ± 0.04; this value increased to 0.71 ± 0.05 after LPS challenge. Phospho-Pyk2 density remained at the same level in TAT-GFP-treated mice challenged with LPS and decreased to 0.37 ± 0.02 in mice pretreated with TAT-Pyk2-CT (p < 0.05 vs. LPS alone).

### Effect of Pyk2 inhibition on pathological change in lung tissues of LPS-induced ALI mice

**Figure 2:**
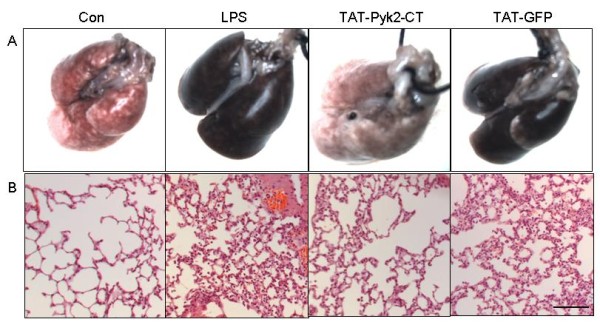
**Effect of Pyk2 inhibition on lung gross anatomy (A) and histology (B) of LPS-challenged mice**. Mice were given an i.p. administration of 10 mg/kg TAT-Pyk2-CT or TAT-GFP 1 h prior to an i.t. administration of LPS. Saline challenged mice were used as negative control (Con). The lung was photographed and prepared for H&E staining 18 h after saline or LPS challenge. Representative image from one of six mice per experimental group was shown. Scale bar: 50 μm.

TAT-Pyk2-CT blocked the characteristic gross anatomic alterations of acute lung injury caused by LPS (Figure 2A). Compared to the pink color for the lungs of saline challenged mice, the lungs of LPS challenged mice were dark red and swollen, indicating lung inflammation and hemorrhage. The lungs of TAT-Pyk2-CT pretreated mice challenged with LPS were similar to those of the saline control mice, while the lungs of TAT-GFP pretreated mice challenged with LPS were similar to the lungs of LPS challenged mice without treatment.

Histological analysis demonstrated that lungs from saline challenged mice had no morphological evidence of injury (Figure 2B). The lungs from LPS challenged mice showed interstitial thickening, alveolar hemorrhage, and cellular infiltration in both interstitial and alveolar compartments, as compared to saline-challenged control mice. TAT-Pyk2-CT pretreatment decreased all of these markers of lung injury, while TAT-GFP pretreatment had no inhibitory effect on lung injury.

### Blockade of lung vascular leakage by TAT-Pyk2-CT

**Figure 3:**
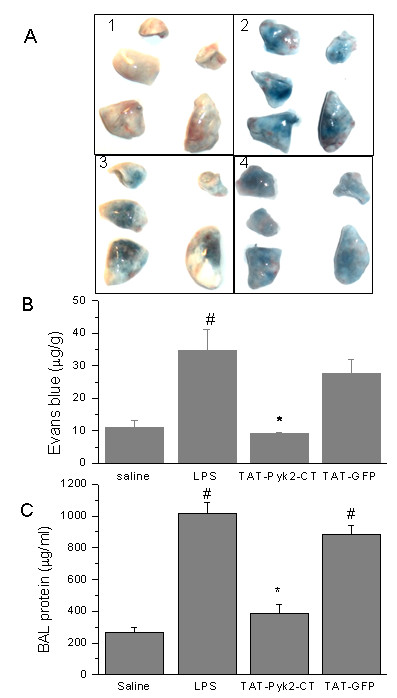
**Effects of Pyk2 inhibition on LPS- induced lung microvascular permeability**. A. Images of the lungs represent Evans blue leakage into lung tissue. Lung vascular permeability was assessed by Evans blue accumulation in the lungs as described in the Materials and Methods. Results are representative of four independent experiments. 1. Saline control; 2. LPS; 3, TAT-Pyk2-CT + LPS; 4. TAT-GFP + LPS. B. Evans blue extravasation was quantified by spectrophotometry. Values are means ± SEM; *n *= 4 per condition. #*P *< 0.05 vs. control group; *P < 0.05 *vs*. LPS alone group. C. Protein concentration was measured in BAL taken from control and LPS-challenged animals. The values presented were the means ± SEMs (*n *= 4 for saline control and 6 each for LPS challenged groups). #*P *< 0.05 vs. control group; *P < 0.05 *vs*. LPS alone group.

LPS induced Evans blue dye accumulation in the lung parenchyma, which was blocked substantially by TAT-Pyk2-CT compared to saline challenged controls (Figure 3A). These results further were confirmed by quantitative analysis of Evans blue-labeled albumin extravasation in the lung preparations (Figure 3B). LPS caused substantial increase in Evans blue accumulation in the lungs (34.8 ± 6.2 μg/g *vs*. 10.9 ± 2.1 μg/g in control animals; *P *< 0.05). Treatment with TAT-Pyk2-CT caused substantial decrease in Evans blue accumulation (9.1 ± 0.5 μg/g vs. 34.8 ± 6.2 μg/g for LPS alone; *P *< 0.05). TAT-GFP pretreatment had no inhibitory effect on Evans blue dye leakage.

TAT-Pyk2-CT also blocks alveolar protein concentration (Figure 3C). In mice receiving LPS alone, BAL protein was increased from 263 ± 33.9 μg/ml for saline challenged control to 1020 ± 64.0 μg/ml (p < 0.05 vs. saline control). Pretreatment with TAT-Pyk2-CT blocked maximal protein concentration in the BAL to 389 ± 53.7 μg/ml (p < 0.05, vs. LPS alone). TAT-GFP pretreatment had no inhibitory effect.

### Effect of Pyk2 inhibition on neutrophilic inflammation during LPS-mediated ALI in mice

**Figure 4:**
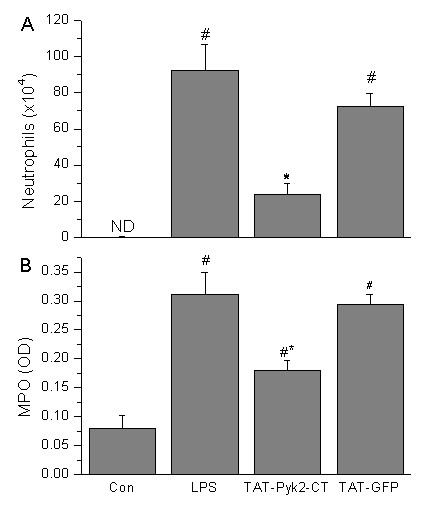
**Effect of Pyk2 inhibition on neutrophil infiltration in alveolar space and lung tissues of LPS-challenged mice**. Mice were given an i.p. administration of TAT-Pyk2-CT (10 mg/kg) 1 h prior to an i.t. administration of LPS. BALF was collected at 18 h following LPS challenge to measure the number of neutrophils (A), and lung tissue homogenates were used to measure MPO activity (B). Saline challenged animals served as controls (con). Each bar represented the mean ± SEM of 4 (saline control) or 6 (LPS challenged groups) mice. #*P *< 0.05 *vs*. control group; *P < 0.05 *vs*. LPS alone group.

TAT-Pyk2-CT caused substantial inhibition of neutrophil infiltration in BAL and lung parenchyma caused by LPS challenge (Figure 4). The baseline BAL neutrophil count was 0.09 × 10^4 ^± 0.08 for the saline challenged control, and increased to 92.2 × 10^4 ^± 14.7 after LPS challenge (Figure 4A). Mice receiving TAT-Pyk2-CT 1 h before LPS challenge reduced neutrophil counts by ~75% (p < 0.05). Control TAT-GFP did not block neutrophil infiltration into the alveolar space.

LPS instillation also caused significant increase in lung MPO activity compared with the control group (Figure 4B). Lung MPO concentration, expressed as optical density (OD) per 150 μg/ml protein, increased to 0.31 ± 0.04 arbitrary units after LPS administration compared with 0.08 ± 0.02 units for saline-treated control mice (Figure 4B; *P *< 0.05). Intraperitoneal injection of 10 mg/kg of TAT-Pyk2-CT 1 h before LPS exposure significantly reduced MPO activity to 0.18 ± 0.02 units compared with the LPS group, while treatment with TAT-GFP had no significant effect.

### Effect of TAT-Pyk2-CT on proinflammatory cytokines in the alveolar space

**Figure 5:**
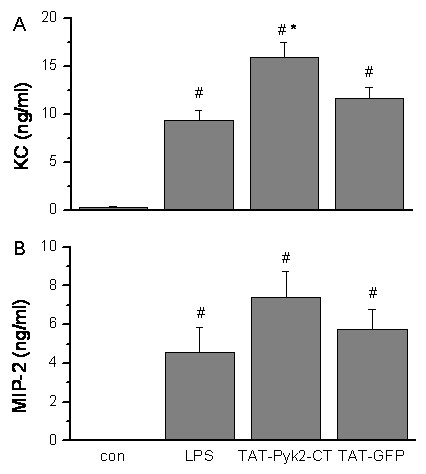
**Effect of Pyk2 inhibition on production of MIP-2 and KC in the BALF of LPS-challenged mice**. Mice were given an i.p. administration of 10 mg/kg TAT-Pyk2-CT 1 h prior to an i.t. administration of LPS. BALF was collected at 18 h following LPS challenge to analyze the concentration of MIP-2 and KC. The values presented were the means ± SEM (*n *= 4 for control, 12 for LPS alone, 9 for TAT-Pyk2-CT + LPS, and 10 for TAT-GFP + LPS). #*P *< 0.05 *vs*. control group; *P < 0.05 *vs*. LPS alone group.

TAT-Pyk2-CT did not block the secretion of chemokines, macrophage inflammatory protein-2 (MIP-2) and keratinocyte-derived chemokine (KC; also called CXCL1 chemokine), two potent known neutrophil-recruiting chemokines [23]. Both chemokines were increased in BAL fluid of mice receiving LPS compared with the saline treated mice (Figure 5A and 5B). LPS induced a 33-fold increase in KC (9.36 ± 1.01 vs. 0.29 ± 0.14 ng/ml) and 470-fold increase in MIP-2 (4.56 ± 1.28 vs. 0.01 ± 0.001 ng/ml), which was not attenuated by TAT-Pyk2-CT.

### Blockade of static lung compliance by TAT-Pyk2-CT

**Figure 6:**
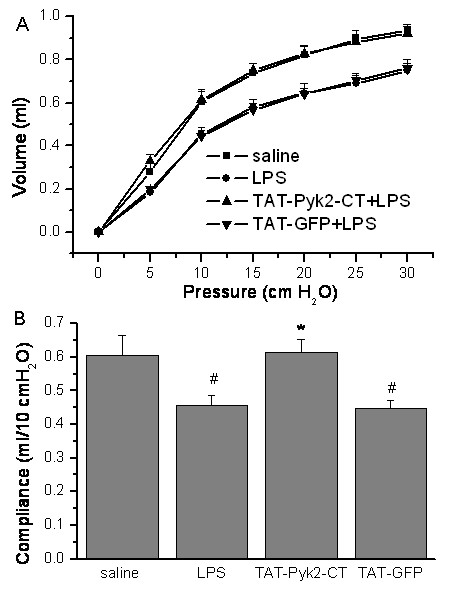
**Effects of TAT-Pyk2-CT on reduced transthoracic static compliance caused by intratracheal administration of LPS**. Mice were pretreated with 10 mg/kg TAT-Pyk2-CT or TAT-GFP 1 h before saline or 10 mg/kg LPS challenge, and (A) pressure-volume (P-V) curves were generated at 18 h after challenge. B. Decrease in transthoracic static compliance caused by LPS treatment. Measurements were means ± SEM at 10 cm H_2_O for each group (n = 6). #*P *< 0.05 *vs*. saline control group; *P < 0.05 *vs*. LPS alone group.

Pretreatment with TAT-Pyk2-CT blocked substantially the decrease in lung compliance caused by LPS. Administration of intratracheal LPS into mice 18 h before measurement caused a right shift of pressure-volume (P-V) curves (Figure 6A) compared with baseline P-V curves generated from saline-challenged mice. Pretreatment with TAT-Pyk2-CT, but not TAT-GFP, significantly blocked the downward shift in Pressure-Volume curve caused by LPS.

Data also were analyzed as lung compliance. *Composite change *in transthoracic static compliance was derived from the linear portion of the P-V curves in each group. Transthoracic static compliance was measured at 10 cm H_2_O (Figure 6B). Static compliance decreased from 0.61 ± 0.06 ml of saline control to 0.46 ± 0.03 ml after LPS treatment (*P *< 0.05 vs. saline control). This reduction in transthoracic static compliance caused by LPS was attenuated to 0.61 ± 0.04 ml in LPS-challenged mice pretreated with TAT-Pyk2-CT (*P *< 0.05 vs. LPS alone group). Treatment with TAT-GFP control did not block the reduction in static compliance caused by LPS.

## Discussion

The objective of this investigation was to examine the role of Pyk2 in mediating ALI caused by LPS in a mouse model in vivo. Our data demonstrate that Pyk2 activation is an important step in the LPS-induction of ALI. We find that TAT-Pyk2-CT effectively blocked the LPS-induced 1) inflammatory cell migration into the lung, 2) protein leakage, and 3) reduction of lung static compliance caused by LPS.

In ALI, the predominant infiltrating inflammatory cells are neutrophils, which play an important role in the pathogenesis in most cases of ALI [24]. When ALI occurs, neutrophils adhere to the capillary endothelium and migrate into the air spaces. Activated neutrophils then release lipid mediators, oxidants, proteases, and other inflammatory mediators, which results in lung injury [25]. As expected, mice exposed to LPS exhibited substantial recruitment of neutrophils into the airways. Treatment with TAT-Pyk2-CT blocked LPS-induced increase in the number of neutrophils in the BAL and lung tissues (Figure 4). Lung histological examination demonstrates that TAT-Pyk2-CT has a significant anti-inflammatory activity during LPS-induced ALI (Figure 2). The infiltration of neutrophils, hemorrhage, and alveolar edema were found to be common and prominent in the LPS group but rare in the TAT-Pyk2-CT pretreated group. These findings may suggest that the protective effect of TAT-Pyk2-CT on ALI caused by LPS was partly attributed to an attenuation of neutrophil infiltration into the lung tissue. The precise extent of neutrophilic effects vs. other causes of increased vascular leak could not be assessed in these studies in vivo. However, investigations in vitro suggest that neutrophils augment vascular leak through increased interendothelial gap formations [26,27].

LPS increases lung barrier permeability [28], which results in alveolar edema and concomitant reduction of lung compliance [29,30]. To quantify the magnitude of pulmonary edema, we measured total protein concentration in the BAL and Evans blue dye extravasation in the lungs. As expected, LPS instillation was found to cause a significant increase in BALF protein concentration and Evans blue dye extravasation into the lungs. LPS-induced increases in these two parameters were inhibited by TAT-Pyk2-CT (Figure 3). Increase in lung barrier permeability in LPS-induced ALI has been previously shown to be neutrophil-mediated [4,24]. Previous studies also have shown that Pyk2 critically regulates the chemotaxis of neutrophils and differentiated neutrophil-like HL60 cells in vitro [11,13]. Pyk2-/- neutrophils have reduced migration on fibrinogen-coated surface and impaired adhesion-dependent release of granular proteins [10]. While these in vitro observations suggest a potential role for Pyk2 involvement in neutrophil mediated lung injury, other potential mechanisms also may be altered by Pyk2 inhibition in vivo. Pyk2 has been identified recently as a potential tyrosine kinase involved in the regulation of VE-cadherin phosphorylation and endothelial junctional integrity [31,32]. Therefore, while the in vivo therapeutic effect of Pyk2 inhibition is demonstrated in these experimental results, the attribution of a single mechanism (i.e. neutrophil mediated endothelial damage) may be insufficient to explain all aspects of vascular leak in LPS-induced ALI.

Proinflammatory cytokines appear in the early phase of an inflammatory response, playing a critical role in ALI development, and contribute to the severity of lung injury [33]. In the present study, we found that LPS induces the production of large amounts of MIP-2 and KC detected in the BAL of LPS treated mice. However, Pyk2 inhibition by TAT-Pyk2-CT did not block the production of KC and MIP-2 in BAL (Figure 5). In fact, KC concentration was increased in BAL of TAT-Pyk2-CT treated and LPS challenged mice. This may be explained by the negative feedback mechanism since TAT-Pyk2-CT treated and LPS challenged mice has less neutrophils in the lung. Anand AR et al. found that inhibition of Pyk2 activity in endothelial cells in vitro by transfection with the catalytically inactive C-terminal Pyk2 significantly blocked LPS-induced IL-8 production [34]. The inability of Pyk2 inhibition to affect IL-8 homolog secretion in mouse BAL in our study may suggest that lung resident cells (such as airway epithelial cells and alveolar macrophages) other than endothelial cells may be at least in part responsible for the secretion of IL-8 homologs found in the BAL. Thus, even in the presence of increased concentrations of chemoattractants in the alveolar space, inhibition of Pyk2 prevented the LPS- induced infiltration of neutrophils into the lung. Similar results have also been observed in mice pretreated with either a p38 inhibitor [35] or JNK inhibitor [36]. Our group and others have reported that Pyk2 inhibition attenuated chemotaxis of neutrophils and HL60 derived neutrophil-like cells to various chemokines [11,13]. Pyk2 inhibition blocked the formation of stable lamellipodia in adherent eosinophils [12,20], which is necessary for subsequent cell migration.

Previous studies have shown that LPS-induced ALI and mortality can be attenuated by pretreating animals with tyrosine kinase inhibitors, although the precise kinase through which these inhibitors exert their effects is not entirely clear [37,38]. Other studies have demonstrated that mice deficient in Src kinase members (hck and fgr double knockouts) are resistant to endotoxin-mediated injury and have reduced neutrophil migration into tissues. These same studies noted that this phenomenon occurred without an effect on serum cytokine levels [39]. A recent study found that the Src family kinase inhibitor PP1 inhibited the LPS-induced increase in neutrophil recruitment and protein leakage [40]. Src family kinases have been implicated in causing maximal Pyk2 activation [41], and were recently found to cause Pyk2 autophosphorylation in T cells [42]. Therefore, it may be reasonable to speculate that these tyrosine kinase inhibitors and Src family kinase inhibitors exert a beneficial anti-inflammatory effect by preventing the full activation of Pyk2.

It is important to note discrepancy between previous reports using mouse neutrophils isolated from bone marrow of Pyk2 knockout mice and human blood neutrophils treated with TAT-Pyk2-CT. Using neutrophils isolated from bone marrow of Pyk2 knockout mice, Kamen et al. found that Pyk2 was required for integrin-mediated degranulation and migration but was not involved in adhesion-induced cell spreading or activation of superoxide production [10]. Using TAT-Pyk2-CT as a Pyk2 inhibitor for human neutrophils, Han et al. suggested that Pyk2 was required for TNF-mediated superoxide release and neutrophil spreading [12]. By contrast, TAT-Pyk2-CT did not affect degranulation of adherent neutrophils or alter neutrophil killing of bacteria. These results, obtained with human neutrophils, obviously differ significantly from observations with Pyk2-deficient murine bone marrow neutrophils. In the current study, we found that neutrophil migration into the lung is blocked by TAT-Pyk2-CT, which also is different from Pyk2^-/- ^mice, showing no defect of neutrophil infiltration to skin pouch in response to subcutaneious *S Aureus *[10]. By contrast, Xu et al. found that mouse neutrophils deficient of non-muscle myosin light chain kinase, which is required for the recruitment and activation of Pyk2, had impaired ability to infiltrate to the lung ex vivo in response to LPS or fMLP [14]. There are many potential explanations for these disparate observations, such as potential compensation for Pyk2 deficiency by overexpression of other kinases, differences in experimental approaches, differences especially between cells from different species, i.e. mature human blood cells and murine bone marrow cells. Likewise, differences in stimulus (S. Aureus vs. LPS) and inflammation loci (skin pouch vs. lung) could also account for disparate outcomes. It has been found that neutrophil infiltration into loci of inflammation is tissue-specific and regulated by different signaling mechanisms [41]. Lastly, off-target effects of TAT-Pyk2-CT could not be excluded.

In summary, we demonstrate that Pyk2 may play a critical role in the pathogenesis of LPS-induced lung injury, and that TAT-Pyk2-CT used as a Pyk2 inhibitor has a protective effect on LPS-induced ALI. Pretreatment with TAT-Pyk2-CT results in a significant reduction in both lung neutrophilia and protein leakage in the BALF. Histological examination also shows that TAT-Pyk2-CT has a significant anti-inflammatory activity during LPS-induced ALI. Since effective anti-inflammatory pharmacotherapy for ALI is still not available, Pyk2 inhibition may represent a potential preventive intervention point in the treatment of ALI.

## List of abbreviations

ALI: Acute lung injury; ARDS: Acute respiratory distress syndrome; BAL: Bronchoalveolar lavage; LPS: Lipopolysaccharide; FAK: Focal adhesion kinase; GFP: Green fluorescent protein; KC: Keratinocyte derived chemokine; MPO: Myeloperoxidase; MIP2: Macrophage inflammatory protein 2; PBS: Phosphate buffered saline; Pyk2: Proline rich tyrosine kinase 2; PV curve; Pressure volume curve; TBS: Tris buffered saline.

## Competing interests

The authors declare that they have no competing interests.

## Authors' contributions

YD carried out the LPS challenge, BAL cell analysis, lung compliance, and lung histology. JL performed the cytokine quantification in BAL. AM performed the Evans blue extravasation study and participated in lung compliance measurement. AL participated in conceiving of the study and revised the manuscript. XZ designed the study, participated in expression and purification of TAT fusion proteins, and drafted the manuscript. All authors approved the final manuscript.
